# Increased expression level of *Dicer* in placenta is associated with the early onset of preeclampsia

**DOI:** 10.18502/ijrm.v21i12.15043

**Published:** 2024-01-25

**Authors:** Fatemeh Mohseni, Mohammad Shekari, Kianoosh Malekzadeh, Pooneh Nikuei, Fatemeh Poordarvish, Ali Atashabparvar, Kourosh Bamdad

**Affiliations:** ^1^Molecular Medicine Research Center, Hormozgan Health Institute, Hormozgan University of Medical Sciences, Bandar Abbas, Iran.; ^2^Pathology Department, Faculty of Medicine, Hormozgan University of Medical Sciences, Bandar Abbas, Iran.; ^3^Department of Biology, Faculty of Sciences, Payame Noor University (PNU), Fars, Iran.

##  Dear Editor,

Preeclampsia (PE) is the most threatening factor for maternal and perinatal health during pregnancy. This syndrome can be diagnosed in pregnant women only through clinical symptoms such as high blood pressure and proteinuria in the second half of pregnancy. So far, no specific biomarkers and predictive tests have been found for the early detection of PE (1).

An association between the expression of microRNAs in the placenta and PE incidence was observed. Many miRNAs are expressed in the placenta while their expression levels are altered in PE (2). Also, the essential factors, including Dicer, Drosha, Argonaute 2, and Exportin 5, which are crucial for miRNA biogenesis, can be found in trophoblast cells (3, 4). Drosha and Dicer, the critical enzymes in miRNA production, produce miRNAs through the sequential processing of long primary RNA transcripts. Dicer indicates a vital role in miRNA maturation (5). The current study aimed to evaluate the expression levels of *Dicer*, as a key enzyme in miRNA biogenesis and processing, in women suffering from mild and severe PE. Expression levels of the *Dicer* gene in PE.

**Figure 1:**
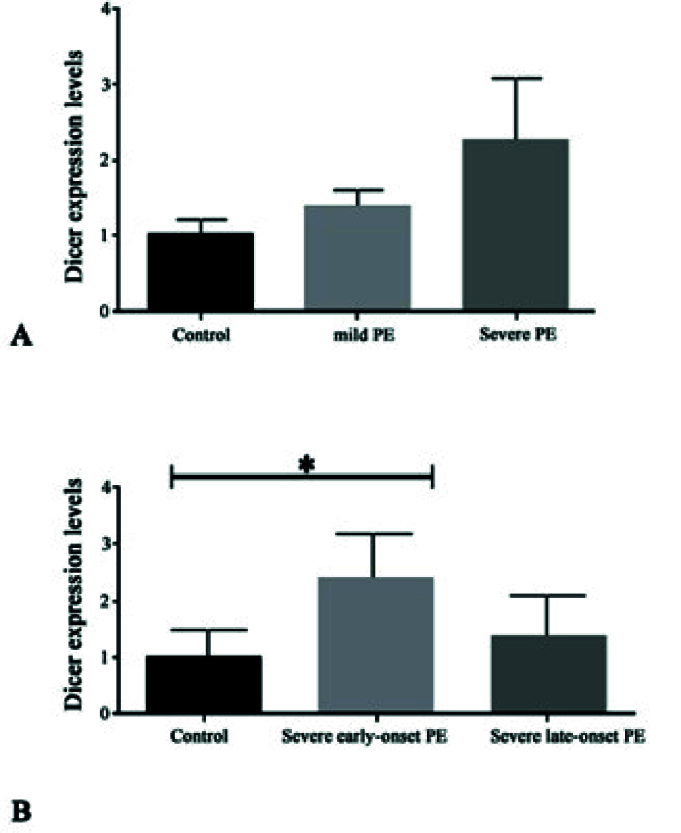
A) Relative expression levels of the *Dicer* gene in different experimental groups. B) Comparison of *Dicer* expression levels in the control group with severe early onset and severe late-onset PE.

In this study, the relative expression levels of the *Dicer* gene in 30 PE samples and 20 control samples were examined using quantitative reverse transcription- polymerase chain reaction. As indicated in figure 1A no significant change in the expression level of the *Dicer* gene was observed in mild PE compared to the control group. In the severe PE group, however, the expression level of *Dicer* seems to be increased, but the difference between these 2 groups was not significant. The data presented in figure 1B showed the upregulation of *Dicer* in severe early-onset PE compared to the healthy group (p < 0.05).

A subgroup of PE samples was also examined using immunohistochemical methods to determine whether mRNA levels reflected protein expression.

**Figure 2:**
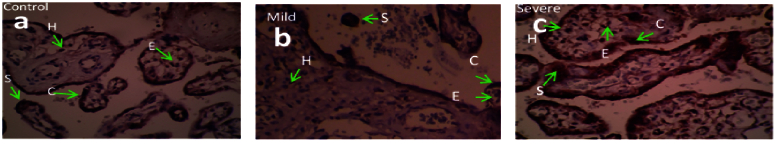
Detection of *Dicer* expression in the placenta of control and different PE samples using immunohistochemistry experiment. C, E, H, and S correspond to cytotrophoblast, endothelial, Hofbauer, and syncytiotrophoblast cells, respectively. The cells were observed with magnification x400.

Immunostaining of placental sections indicated that *Dicer* was expressed in all placental cells (syncytiotrophoblast, cytotrophoblast, endothelial cells, and Hofbauer cells) in both PE and controls. The participants affected by severe and early onset PE displayed increased staining intensity, predominantly in syncytiotrophoblast and cytotrophoblast cells. The mild PE samples showed moderate staining and in controls, mild staining was observed (Figure 2). The relative staining intensity of different cells was quantified, and severe PE showed increased *Dicer* expression compared to other experimental groups.

In the current study, the expression levels of *Dicer *gene were evaluated in healthy control and different stages of PE using qRT-PCR and immunohistochemistry methods. As shown in figure 2, expression of *Dicer* in the transcription level increased in the early onset of severe PE compared to control samples and other PE stages. Furthermore, the expression of *Dicer* in the translation state has been evaluated using immunohistochemistry experiment, and its expression was also found to be increased in the early onset of severe PE (Figure 2).

During the normal development of the placenta, many miRNA molecules are expressed in the human placenta which display an essential role in physiological processes including differentiation, proliferation, angiogenesis, migration, invasion, apoptosis, and metabolism of placental cells development, particularly trophoblast cells (6). A previous study reported upregulation of miR-21-5p and miR-155-5p miRNA molecules in preeclamptic pregnancies, especially in severe and late-onset PE subgroups compared to healthy pregnancies (7). In our study, increased expression of *Dicer* in severe PE may explain the overexpression of miR-21-5p and miR-155-5p miRNAs in PE. Overexpression of miR-210 in PE can suggest it as a promising biomarker for monitoring pregnancy. Overexpression of this molecule displays a negative effect on some cell processes, such as cell migration and trophoblast invasion (8).

Key molecules such as Dicer and Drosha are involved in the process of miRNA biogenesis and maturation. Previous studies have indicated that the knockdown of Dicer induces cell proliferation, apoptosis, angiogenesis inhibition, reduced endothelial cells migration, and loss of miRNA mature sequence (9). Based on a previous study, the expression levels of *Drosha, *another member of miRNAs-processing proteins, were found to be reduced in the preeclamptic placentas (10). In contrast, our study indicated upregulation of *Dicer* in the early onset of severe PE. Since the production of miRNAs is a complex process and various enzymes are involved in it, the overexpression of *Dicer* in the early onset of severe PE cannot be considered as a predictive biomarker for PE. Therefore, the expression of various proteins involved in miRNA processing as well as the expression levels of different miRNAs should be studied in detail.

The results of this study indicated overexpression of *Dicer* in the early onset of severe PE. Regarding the importance of miRNA molecules in placental development, the RNase III enzymes such as Dicer which drives miRNA biogenesis should be investigated for their role in PE pathogenesis to shed light on some aspects of this phenomenon.

###  Acknowledgments

This work was financially supported by Research Vice-Chancellor of Hormozgan University for Medical Science, Hormozgan University of Medical Sciences, Bandarabbas, Iran (grant number: 93122).
